# Targeting Cellular Lipid Rafts for Dynamic Nuclear Polarization Nuclear Magnetic Resonance

**DOI:** 10.1002/cbic.202600001

**Published:** 2026-02-28

**Authors:** Sarah A. Overall, Agnes Eck, Ancy T. Wilson, Dorothea Pinotsi, Sina J. Hartmann, Katja Packebusch, Snorri Th. Sigurdsson, Alexander B. Barnes

**Affiliations:** ^1^ Institute of Molecular Physical Sciences ETH Zurich Zurich Switzerland; ^2^ Science Institute University of Iceland Reykjavik Iceland; ^3^ Scientific Center for Optical and Electron Microscopy ETH Zurich Zurich Switzerland

**Keywords:** hyperpolarization, in‐cell dynamic nuclear polarization‐nuclear magnetic resonance, lipid raft, solid‐state nuclear magnetic reosnance, targeted dynamic nuclear poarization

## Abstract

Lipid rafts serve as important platforms for membrane and signaling proteins. The mechanisms underlying the targeting of nonmyristylated drugs and proteins to lipid rafts remain poorly understood, and the specific structural interactions that govern their localization and stabilization within these membrane microdomains are unclear. This is largely due to a lack of techniques with angstrom resolution that are capable of investigating these membrane microdomains. In‐cell nuclear magnetic resonance (NMR) spectroscopy is the only technique with the potential for obtaining such information. Here, we introduce an approach to investigate lipid rafts by in‐cell dynamic nuclear polarization (DNP) NMR by covalently linking the polarizing agent AsymPol to the raft‐specific protein Ostreolysin A (OlyA). We demonstrate the method's specificity via fluorescence microscopy and obtain DNP enhancements even at very low concentrations of spin‐labeled OlyA, whose heterogeneous localization can be identified in DNP buildup curves. Through this work, we identify cross‐effect efficiency as a key limiting factor in the pursuit of lipid raft‐targeted DNP, revealing important areas of development for enabling targeted DNP of these important cellular structures.

## Graphical abstract

Illuminating lipid rafts within intact cells using dynamic nuclear polarization (DNP). Targeting lipid rafts with the protein ostreolysin A allows the selective delivery of DNP‐compatible radicals, generating hyperpolarization of nuclear spins in the lipid raft.

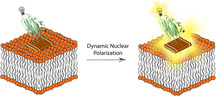

## 1 Introduction

Lipid rafts are dynamic, ordered domains within cell membranes, enriched in cholesterol and sphingolipids, and often characterized as detergent‐resistant [1, 2, 3, 4, 5]. Rafts provide platforms for the regulation of cell signaling through the spatial segregation of protein–protein interactions. The dynamic regulation of lipid raft organization and the recruitment of proteins to lipid rafts plays a critical role in proper immune receptor signaling [5, 6], in growth factor responses contributing to oncogenesis [5, 7, 8, 9], and in viral entry, such as in HIV infection [10], making the study of lipid rafts an active field of significant clinical interest. However, the study of these domains on the atomic scale is challenging due to a lack of experimental techniques that can specifically identify and observe these domains within intact cells.

In‐cell dynamic nuclear polarization (DNP) nuclear magnetic resonance (NMR) is an emerging technique for the characterization of membrane‐bound cell signaling structures and large cellular assemblies within their native cellular context [11, 12, 13, 14]. DNP provides large sensitivity gains through nuclear hyperpolarization, where polarization is transferred from electrons to nuclei [15, 16, 17, 18]. Conventionally, a homogeneous distribution of the polarizing agent throughout the sample is crucial to achieve optimal enhancements [19], maximizing the distribution of polarization through the bulk of the sample. However, this increases the complexity of the NMR spectra, making interpretation difficult. As NMR represents the ensemble average of a sample, this not only complicates the spectra of cells but also makes the interpretation of the relative contribution of different organelle structures to spectral resonances challenging. In complex and heterogeneous samples, such as cells, the selection of the sites of interest can greatly improve spectral resolution. Therefore, targeted DNP is ideally suited to extracting localization‐specific structural information from within cells.

Targeted DNP has been used to specifically target polarizing agents to proteins and other biomolecules of interest. So far, targeted DNP has been achieved by employing site‐directed spin‐labeling, either directly on a protein of interest [20, 21, 22, 23] or its ligands [24, 25, 26]. Furthermore, paramagnetic metal ions have been investigated for targeted DNP on nucleic acids [27] and as spin‐labels for proteins [21]. However, to our knowledge, no system has been described for targeted DNP on lipid rafts.

A range of molecules have been identified that specifically target lipid rafts, such as Cholera Toxin subunit B (CT‐B) [28] and anti‐GM1 antibodies, both of which bind to the lipid, ganglioside mono‐sialic acid 1 (GM1), enriched in lipid rafts [29, 30], or cholesterol‐binding reagents such as Filipin [31]. However, cholesterol and gangliosides are also found outside of the ordered domains of lipid rafts, albeit at lower concentrations. A more specific marker for lipid rafts is the complex between cholesterol and sphingomyelin. A range of fungal toxins, in particular ostreolysin A (OlyA), which specifically targets the complex of cholesterol with sphingomyelin, is only found in lipid rafts [32, 33]. OlyA is part of a cytolytic pore‐forming complex, whose cytolytic activity requires pleurotolysin B, produced by *Pleurotus ostreatus*, but is nonlytic in its absence [34, 35, 36]. OlyA has been used for extracellular fluorescent labeling of lipid rafts in both live and fixed cells [32, 36]. Here we employ maleimide chemistry‐based labeling strategy to conjugate AsymPol to the C‐terminus of a cysteine mutant of OlyA [37]. This approach provides a tool to specifically enhance lipid raft regions in cryogenically preserved cells in suspended animation with in‐cell DNP‐NMR spectroscopy. Additionally, we probe the specificity of OlyA to lipid rafts using confocal fluorescent microscopy by co‐staining with GM1 on live cells. The effective implementation of this technique holds promise for elucidating numerous unresolved inquiries concerning processes that involve lipid rafts on the atomic scale.

## 2 Results and Discussion

### 2.1 Synthesis of Maleimide AsymPol and AF647‐OlyA

**FIGURE 1:**
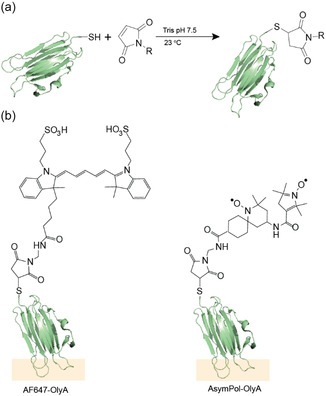
Conjugation of tracking molecules to OlyA. (a) Conjugation of OlyA to maleimide‐modified molecules in 50 mM Tris buffer at pH 7.5, 150 mM NaCl at room temperature. (b) Chemical structures of AF647 and AsymPol tagged OlyA. The orange region highlights the part of OlyA that interacts with the sphingomyelin:cholesterol complex.

To create a polarizing agent for targeting lipid rafts for in‐cell DNP NMR, we recombinantly prepared OlyA with a C‐terminal free cysteine modification, as previously described [32]. The C‐terminal cysteine enables conjugation of OlyA to both maleimide‐modified Alexa Fluor 647 (AF647) for fluorescence microscopy and maleimide‐modified AsymPol Site‐Directed Spin‐Label (AsymPol‐M4‐SDSL; SI‐1), for targeted in‐cell DNP (Figure 1a,b). The conjugation of maleimide AF647 to purified OlyA was monitored b Sodium‐Dodecyl‐Sulphate PolyAcrylamide Gel Electrophoresis‐(SDS‐PAGE), where the conjugated AF647‐OlyA results in a 1 kDa shift in the observed molecular weight (SI‐2). We observed around 80% conjugation after 3 h at room temperature, confirmed by absorption spectroscopy at 280 and 651 nm utilizing the respective extinction coefficients of OlyA and AF‐647. We were unable to monitor the conjugation of AsymPol‐M4‐SDSL to OlyA by SDS‐PAGE, because the molecular weight difference between OlyA and AsymPol‐OlyA is too small. Therefore, we used electron paramagnetic resonance (EPR) spectroscopy to determine the coupling efficiency of the biradical to OlyA (SI‐3).

### 2.2 Localization of AF647‐OlyA in JLat 9.2 T Cells

**FIGURE 2:**
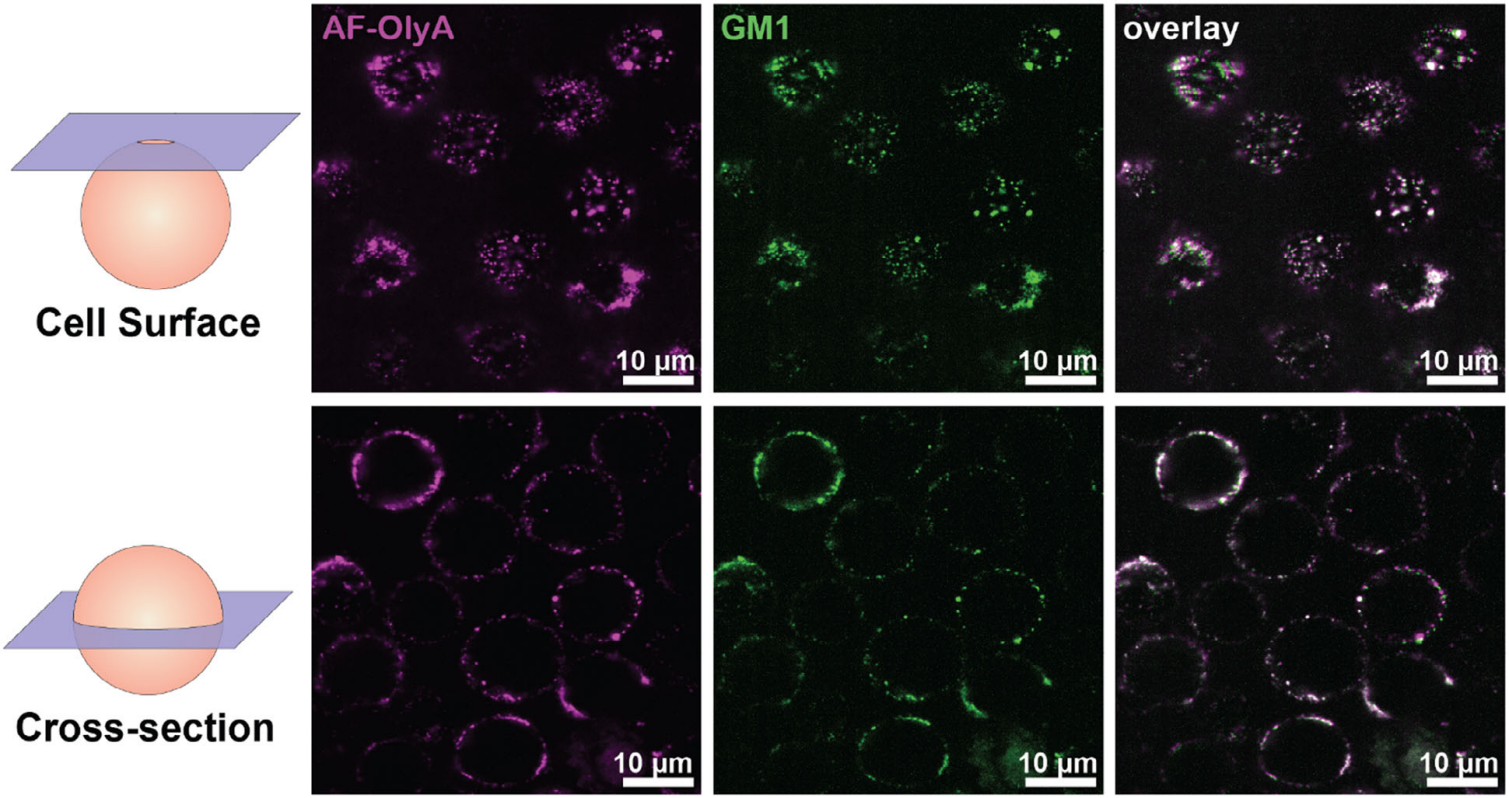
Confocal microscopy of OlyA‐AF647‐stained JLat 9.2 T cells. JLat 9.2 T cells were costained with recombinantly produced OlyA‐647 (magenta) and anti‐GM1‐CF568 antibodies (green). Cells were imaged using a Nikon Ti2 microscope equipped with a confocal re‐scan module. The top panels are cells imaged on the surface, and the bottom panels are the same cells imaged at the cell cross‐section (middle plane of the cells).

We determined the localization of AF647‐OlyA on JLat 9.2 T cells by confocal microscopy. A perfect colocalization of AF647‐OlyA with the lipid raft marker GM1 was observed (Figure 2). No internalization of AF647‐OlyA was seen after 15 min of incubation and after 30 min of live cell imaging, which allows for at least 45 min of sample preparation for DNP to ensure that radical targeting is specific to plasma membrane lipid rafts. Our findings are congruent with what has been reported before on OlyA‐stained MDCK (Madin‐Darby Canine Kidney) cells [32, 38].

### 2.3 In‐Cell DNP‐NMR of AsymPol‐OlyA Labeled Mammalian T Cells

In‐cell DNP‐NMR experiments provide a static, low‐temperature snapshot of membrane organization that reflects the compositional heterogeneity present at the time of freezing. The stabilization of ordered membrane domains, such as lipid rafts, at low temperatures may result in coalescence and enlargement of raft domains during flash freezing [39, 40]. Furthermore, there are indications that protein association with lipid rafts can be altered upon freezing, though this is largely studied by western blotting of detergent‐resistant membranes extracted from cells [41]. Furthermore, these studies expose cells to prolonged low temperatures (0–4°C). The effects of 1–10 min of low temperature exposure prior to complete freezing of the membrane are unclear. However, the AsymPol‐OlyA is a tool that could be used to probe the structure and organization of lipid rafts under cryogenic conditions, and may become an important tool for the study of cryopreservation of sperm and blastocysts used in IVF (in vitro fertilization) procedures and the preservation of blood cells. The DNP capabilities of this reagent were therefore investigated following confirmation of the localization of AF647‐OlyA to surface lipid rafts.

**FIGURE 3:**
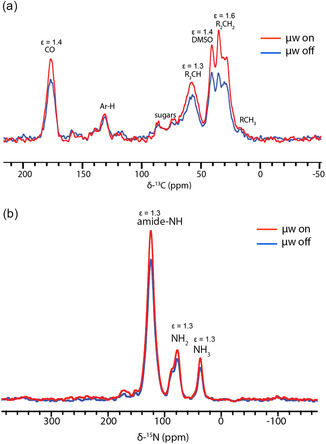
Cross‐polarization spectra of DNP‐enhanced mammalian cells with AsymPol‐OlyA. (a) Natural abundance ^13^C JLat 9.2 T cells labeled with 0.5 nmol/million AsymPol‐OlyA. (b) ^15^N labeled HEK 293T cells labeled with 0.5 nmol/million AsymPol‐OlyA. Microwave on (red line) and microwave off (blue line). Spectra were acquired at 9.4 T with 9 kHz MAS at 86 K (microwaves off) or 91 K (microwaves on) and 128 transients.

JLat 9.2 T cells were labeled with freshly conjugated AsymPol‐OlyA at 0.5 nmol OlyA/million cells for 15 min prior to cryogenic preservation for in‐cell DNP‐NMR. Overall enhancements were *ε* = 1.3–1.6 for ^13^C (Figure 3a). We also analyzed enhancements of ^15^N on the surface of ^15^N‐labeled Human Embryonic Kidney (HEK) 293T cells (since ^15^N‐RPMI was not available), as natural abundance ^15^N was not detectable in unlabeled JLat 9.2 T cells. Here, we also observed enhancements of 1.3 (Figure 3b). These enhancements are small but expected, given that plasma membrane localized lipid rafts account for around 0.2% of the total sample volume based on analysis of the surface occupancy of lipid rafts from Figure 2 and an average cell radius of 5.0 µm.

With such small enhancements, we presume that locally hyperpolarized spins experience a large enhancement of the order of *ε* ≈ 30, as reported for AsymPol at 9.4 T [42], and that much of the enhancement is attributable to the enhancement of only a small number of locally polarized spins. Therefore, we determined the potential for detecting localized enhancements by co‐labeling JLat 9.2 T cells with a 1:1 mixture of AsymPol‐OlyA and ^15^N‐OlyA (that contains no radical and a quenched C‐terminal cysteine), with the intention of detecting lipid raft‐associated ^15^N‐OlyA spins. However, we were unable to detect any ^15^N signal (SI‐4), indicating that direct detection of directly hyperpolarized spins associated with lipid rafts is below the detection limit of our system. Furthermore, this 50% dilution of the loaded radical produced similar global enhancements, suggesting that the cells are loaded to saturation with AsymPol or that the global enhancements are too low to detect appreciable differences in the local enhancement.

We then investigated whether localized hyperpolarization could be detected indirectly through the measurement of buildup times (*T*_B_). Since we can measure AsymPol‐OlyA‐mediated enhancements, we hypothesized that the buildup times might be measurably faster when microwaves are on compared to the buildup time measured in the absence of microwaves; this would be the manifestation of the targeted localization of AsymPol‐OlyA, observed by confocal microscopy, in the DNP‐NMR data according to the core–shell spherical geometry model proposed and modeled by Emsley and colleagues [43, 44]. JLat 9.2 T cells do not exhibit specific signals associated with intracellular components in the ^13^C spectrum, according to the model described by Pinon A et al. [43] Thus, we cannot distinguish source and target signals but can only monitor the influence of microwaves on the build‐up of the combined signals.

**FIGURE 4:**
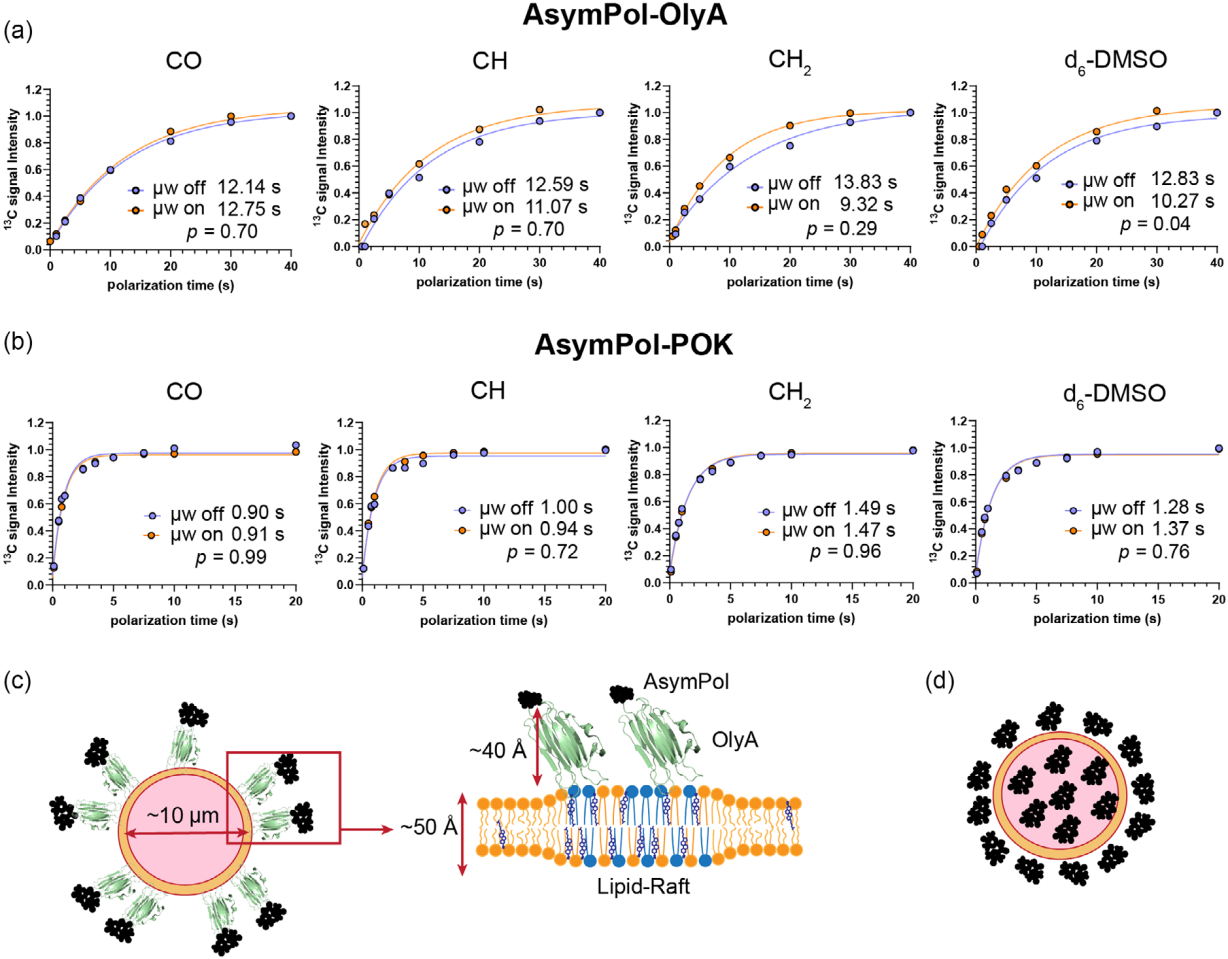
T_B_ buildup curves of ^13^C signals in unlabeled JLat9.2 T cells under DNP conditions. (a) *T*_B_ buildup curves with AsymPol‐OlyA (1 nmol/million cells). (b) *T*_B_ buildup curves with Asympol‐POK (5 mM or 5 nmol/million cells). Data were acquired using a saturation recovery experiment at 9.4 T with 9 kHz MAS. (c) Model of a ‘core–shell’ distribution of AsymPol (black structures) in OlyA labeled cells. (d) Comparative model of a homogeneous distribution of AsymPol‐POK in cells.

We measured *T*_B,on_ and *T*_B,off_ by saturation recovery experiments in the presence or absence of microwaves. We observed small increases in *T*_B_ when microwaves were on when cells were labeled with AsymPol‐OlyA (Figure 4a), indicative of a spatial separation of AsymPol‐OlyA in at least parts of the sample. The shortened buildup time with microwaves on can be attributed to the additional contribution to the buildup time by fast polarization buildup of hyperfine‐coupled spins in the vicinity of AsymPol, shortening the effective buildup time of nuclei in the bulk. This is expected because we know from confocal microscopy that the radical is indeed spatially segregated and confined to the cell surface. As a comparison, we measured the buildup behavior of JLat 9.2 T cells prepared with a relatively homogeneously distributed radical, AsymPol‐POK. In the case of AsymPol‐POK, we obtained an enhancement of 43 and see no difference in the build‐up of polarization when microwaves are on or off (Figure 4b), indicating a homogeneous distribution of radicals in which the buildup time is dominated by paramagnetic relaxation effects that are independent of microwave irradiation. This is further supported by the relatively good fit of a monoexponential function to the AsymPol‐OlyA buildup data, suggesting that the buildup is likely dominated by nuclei at the surface of the cell in close proximity to the radical, with a small contribution of the uncoupled bulk. However, we are clearly at the limits of detection of such phenomena.

The data indicate that we can indeed indirectly detect the small population of directly hyperpolarized spins associated with lipid rafts as a component of the buildup time. Though the lack of raft‐specific resonances in unlabeled cells prevents the direct measurement of the local enhancement, the AsymPol‐OlyA reagent provides an opportunity for structural studies of proteins and small molecules that localize to lipid‐rafts if they are site‐specifically labeled with spectrally resolved isotopes such as ^19^F. The fact that we observe an increase in the effect with microwaves as the degree of substitution of the carbon spin decreases and only in AsymPol‐OlyA labeled cells further supports this notion. For a proton‐dense solid, such as cells, we expect the distribution of spin‐polarization to occur radially with an estimated ^1^H spin diffusion coefficient of ∼1 × 10^5^ Å^2^ s ^−1^ [45]. With this estimation and a diffusion length of around 110 nm, assuming a *T*_B_ of 12 s. This would indicate that polarization does not penetrate far into the cell, which is on average 5 µm in radius, resulting in only ∼ 4% of the cell volume (and therefore sample volume) being polarized, consistent with the monoexponential behavior of the buildup curves with AsymPol‐OlyA. Thus, the small enhancements observed can be attributable to the low loading of cells with radicals, simply due to the limited number of OlyA binding sites per cell. With an estimated 1.2 nm^2^ binding area and 20% of the plasma membranes surface constituting 100 nm lipid‐rafts, 1–2 million OlyA molecules are expected to bind to each cell. From this, the local radical concentration is expected to be 1–3 mM for > 80% radial conjugation. Enhancements of *ε* ≈ 30 have been reported with 10 mM AsymPol [42]. If we now estimate the local enhancement using the following formula:

$$\varepsilon_{\text{global}}=1+\left(\varepsilon_{\text{local}}-1\right)3\frac{L}{r}$$

where $\varepsilon_{\text{global}}$ is the measured enhancement, $\varepsilon_{\text{local}}$ is the enhancement within the vicinity of the radical, *r* is the radius of the cell, and *L* is the diffusion length estimated from the *T*_B_ curves. With a global enhancement of 1.4, a spin diffusion length of 110 nm, and a cell radius of 5 µm, the local enhancement is ∼ 7. Conversely, a local enhancement of 150 would produce a global enhancement of 10. Together, this suggests that site‐specific enhancement of highly localized cellular regions like lipid rafts is possible and feasible, but will predominantly be limited by the local enhancement. The primary determinant of the local enhancement will be the cross‐effect efficiency of the biradical used and local radical concentration. The design of clickable radicals with higher cross‐effect efficiencies and increasing the number of labeling sites on OlyA may be able to overcome the sensitivity issue that arises when attempting to polarize such a small number of total spins. Furthermore, advances in microwave delivery, alternative hyperpolarization methods, and even ultra‐low temperature DNP will continue to improve the sensitivity and spatial discrimination of localized DNP‐NMR.

Such developments will make for the possibility of correlative workflows. Optical methods enable visualization of membrane‐protein localization with high spatial resolution in individual cells, which primarily reports on spatial distribution and organization rather than molecular structure or chemical composition. Complementary to this, localized in‐cell DNP‐NMR offers the potential for obtaining composition and structural information of membrane environments, molecular order, and intermolecular interactions, with sensitivity preferentially enhanced for molecules in close proximity to the polarizing agent or binding site. In addition to sensitivity gains, differences in hyperpolarization buildup kinetics may provide indirect information on spatial confinement and domain organization through established spin diffusion models, yielding model‐dependent estimates of characteristic domain dimensions. In this context, the technique is a complementary approach that links spatial localization to molecular‐level structural and chemical insight across whole cell populations. Thus, the establishment of such complementary and correlative spectroscopy toward the atomic and nanoscale characterization of complex cellular structures is a significant motivation for this work.

## 3 Conclusion

We have shown that the biradical polarizing agent AsymPol can be targeted to lipid rafts found in plasma membranes of intact cells through conjugation to the OlyA protein. We demonstrate an enhancement of up to 1.6 at 9.4 T. We additionally identify an important limitation for in‐cell targeted DNP when utilizing the current class of polarizing agents that have been optimized for a homogeneous distribution, namely the limited number of cellular sites that can be targeted. This strictly limits the radical loading of cell samples with these reagents, which we identify as the dominant source of the low enhancements observed with this approach. Despite this limitation, we can still demonstrate heterogeneous polarization build‐up in AsymPol‐OlyA‐labeled cells compared to homogeneously distributed AsymPol‐POK radicals, indicative of the localization of the radical. Thus, efficient targeted DNP of small cellular structures that constitute a small percentage of the sample volume will require a significant improvement in the cross‐effect efficiency of clickable radicals.

## Supporting Information

The authors have cited additional references within the Supporting Information [46]. **Supporting Fig. S1**: Characterization of AsymPol‐M4‐SDSL. **Supporting Fig. S2:** Coupling efficiency of OlyA to AF647**.** 15% Tris‐Tricine SDS‐PAGE of purified OlyA and AF647 conjugated OlyA. **Supporting Fig S3:** EPR spin counting. **Supporting Fig. S4:** Spectra of ^15^N‐labeled OlyA bound to JLat 9.2 T cells with unlabeled AsymPol‐OlyA.

## Conflicts of Interest

The authors declare no conflicts of interest.

## Supporting information

Supplementary Material
