# Supplementary material for: Targeting Cellular Lipid Rafts for Dynamic Nuclear Polarization Nuclear Magnetic Resonance
Source: Chembiochem. 2026 Feb 28;27(5):e202600001. doi: 10.1002/cbic.202600001 (PMC12949625; doi:10.1002/cbic.202600001)
Supplement: Supplementary file 1 — Supplementary Material [file CBIC-27-e202600001-s001.pdf]

Supporting Information

Targeting cellular lipid rafts for dynamic nuclear polarization NMR

Sarah A. Overall*[a], Agnes Eck[a], Ancy T Wilson[b], Dorothea Pinotsi[c], Sina J Hartmann[a], Katja Packebusch[a], Snorri Th. Sigurdsson[b] and Alexander B. Barnes*[a]

Table of Contents

Supporting Information 1: Experimental Methods……………………………………………………………1

Supporting Information 2: Coupling efficiency of OlyA to AF647……………………………..…………..…4

Supporting Information 3: EPR spin counting…………………………………………………………..……..…4

Supporting Information 4: Spectra of ^{15}N-labeled OlyA bound to JLat 9.2 T cells with unlabeled AsymPol-OlyA…………………………………………………………………………………………………..………5

References…………………………………………………………………………………………….………5

Supporting Information 1: Experimental Methods

Expression and Purification of OlyA

Ostreolysin-A was expressed and purified as described[33]. Briefly overnight cultures of OlyA transformed BL21(DE3)pLysS *E.coli* were grown in luria broth overnight at 37 °C with 50 µg/mL ampicillin. The culture was diluted to 1 L and grown at 37 °C until OD_{600} = 0.6 then induced with 1 mM IPTG and grown at 18 °C for 16 h. The cells were then harvested by centrifugation at 10,000 g for 30 min. The cell pellet was resuspended in lysis buffer (50 mM Tris pH 8.3, 100 mM NaCl, lysozyme, DNAase, 1 mM PMSF, 2 mM EDTA and 0.1% TritonX-100 and 1 mM TCEP) and processed 5 times through a microfluidizer. The lysate was clarified by centrifugation at 10,000 g for 30min at 4°C. The lysate was then passed through a 10 mL His-Trap column equilibrated in 50 mM Tris pH 7.5, 150 mM NaCl, 50 mM imidazole and 1 mM TCEP. OlyA was eluted with 500 mM imidazole. Protein containing fractions were collected, concentrated and purified by size exclusion chromatography using a Superdex 75 26/60 column with 50 mM Tris pH 7.5, 150 mM NaCl and 1 mM TCEP.

Synthesis of AsymPol-M4-SDSL

All commercially available reagents were purchased from Sigma-Aldrich, Inc. and used as received. DMF was dried over CaH_2 and freshly distilled before use. All moisture- and air-sensitive reactions were carried out in oven-dried glassware under an inert atmosphere of argon. Analytical thin-layer chromatography (TLC) was carried out using glass plates precoated with silica gel (0.25 mm, F-254, Silicycle) and compounds were visualized under UV light as well as staining with phosphomolybdic acid. Column chromatography was carried out using 230−400 mesh silica gel (F60, Silicycle). Radicals show broadening and loss of NMR signals due to their paramagnetic nature and, therefore, these NMR spectra are not shown. CW-EPR spectra were recorded on a MiniScope MS200 spectrometer (Magnettech Germany). Mass spectrometric analyses of all organic compounds were carried out on a high-resolution mass spectrometer with electrospray ionization (ESI-HRMS, Bruker, MicrOTOF-Q) in positive or negative ion mode. The purity of all radicals were ascertained on Macherey-Nagel Nucleodur C18 Pyramid 4.6 × 150 mm analytical column with UV detection at λ = 254 nm on Agilent 1200 HPLC system, with a flow rate of 0.75 mL/min using the following gradient: Solvent A, 0.1% TFA in water; solvent B, CH_3CN; isocratic 4% B for 4 min, 26 min linear gradient to 100% B, 4 min isocratic 100% B, 2 min linear gradient to initial conditions, where it was run for additional 4 min.

Abbreviations: BOP, benzotriazol-1-yloxytris(dimethylamino)phosphonium hexafluorophosphate; DIPEA, N,N-diisopropylethylamine; HOBt, hydroxybenzotriazole; DMF, dimethylformamide; EtOAc, ethyl acetate; pet. ether, petroleum ether; MeOH, methanol.

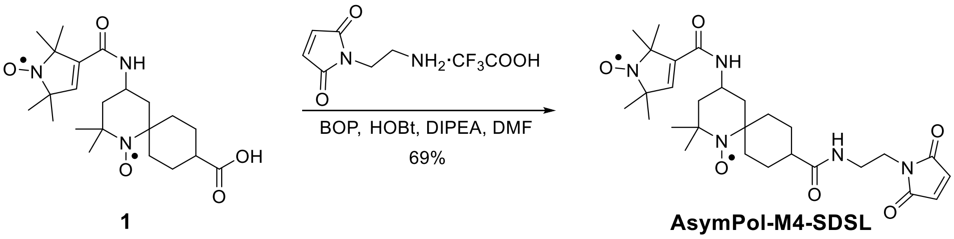

1 **AsymPol-M4-SDSL**

AsymPol-M4-SDSL: To a stirred solution of compound **1**[37] (28 mg, 0.07 mmol) in DMF (0.4 mL) were added BOP (44 mg, 0.1 mmol), HOBt (14 mg, 0.1 mmol) and DIPEA (35 µL, 0.3 mmol) at 22 °C, followed by N-(2-aminoethyl)maleimide trifluoroacetate salt (25.3 mg, 0.1 mmol). The reaction mixture was stirred for 2 h, satd. $NaHCO_3$ (10 mL) was added, and the reaction mixture was extracted with CH_2Cl_2 (3 x 10 mL). The combined organic phases were dried over Na_2SO_4 and the solvent was removed under reduced pressure. The residue was purified by column chromatography using gradient elution (EtOAc:pet. ether 50:50 to MeOH:CH_2Cl_2 5:95) to yield **AsymPol-M4-SDSL** (25 mg, 69% yield) as an orange solid. TLC (Silica gel, MeOH:CH_2Cl_2 0.5:9.5), Rf = 0.4 ESI-HRMS (m/z): calcd. for $C_{28}H_{41}N_5O_6$ $[M+Na]^+$ 566.2949, measured 566.290

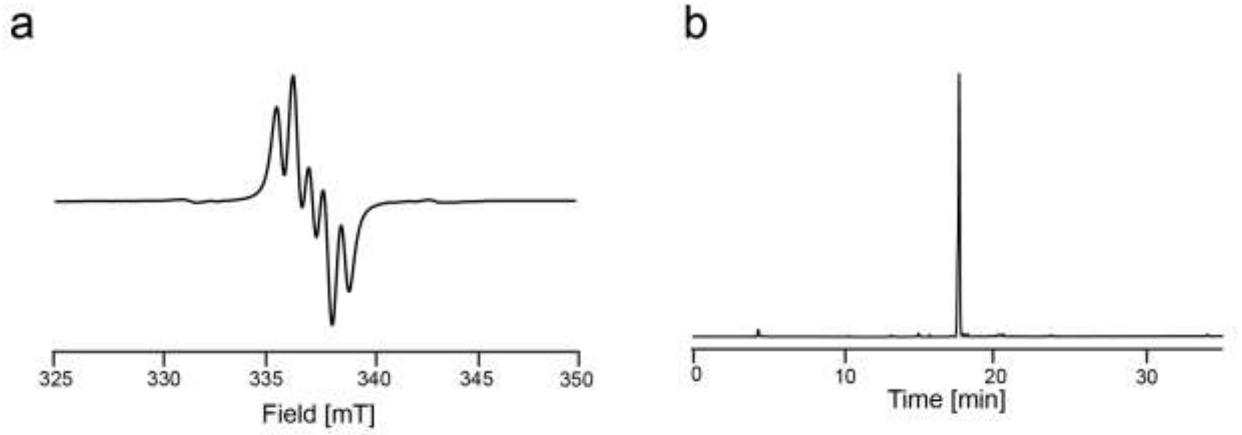

Figure 1. Characterization of AsymPol-M4-SDSL. a) EPR spectroscopy of purified AsymPol-M4-SDSL in MeOH. b) HPLC trace of purified AsymPol-M4-SDSL with CH_2Cl_2 as mobile phase.

Coupling Alexa Fluor 647 to OlyA

OlyA previously purified in 50 mM Tris pH 7.5, 150 mM NaCl and 1 mM TCEP was buffer exchanged to remove the TCEP using a PD-10 desalting column. To 50 µM OlyA a 10-fold excess of Alexa Fluor 647 C2 Maleimide was added and left to react at 4 ◦C for 72 hours. Subsequent SDS page analysis on 12% Tris-glycine gels suggested near-complete coupling. The unreacted maleimide was removed by size exclusion chromatography on a Superdex 75 10/300 column equilibrated in 50 mM Tris pH 7.5, 150 mM NaCl and wrapped in aluminum-foil. Absorption spectroscopy at 280 nm and 651 nm on a NanoDrop One C suggested that 75% of protein was labeled. All steps were carried out in the dark and reaction tubes were wrapped in aluminum foil to prevent bleaching of the fluorophore.

Coupling AsymPol to OlyA

To 50 µM buffer exchanged OlyA, a 10-fold excess of AsymPol-M4-SDSL was added and left to react at room temperature for 3 hours under moderate agitation. AsymPol-OlyA was subsequently purified by size exclusion chromatography on a HiLoad 26/600 Superdex 75 pg column equilibrated in degassed 50 mM Tris pH 7.5 and 150 mM NaCl. Spin-labeling efficiency was determined by CW EPR.

DNP solid-state NMR spectroscopy

All DNP solid-state experiments were acquired on a 400 MHz (9.4 T) wide bore Bruker spectrometer equipped with an HXY low temperature MAS probe operating at 86 K microwaves off and 91 K microwaves on and 9 kHz MAS. Microwaves were provided by a mounted Klystron with 5.1 W output. Cross polarization was achieved using a 70% linear ramp with ^1H spin locking frequency of 59k Hz and ^{13}C spin lock at 50 kHz. T_B curves were generated with saturation recovery experiments on both ^1H and ^{13}C channels followed by echo detection and 96 kHz

SPINAL64 decoupling over acquisition and 1028 scans. All experiments were acquired with a recycle delay of 25 s.

Data was processed in topspin 4.4.0. T_B build up curves were determined by plotting peak intensity as a function of polarization delay and fitting to equation 1 in GraphPad Prism.

$$I(t) = I_0\left(1 - e^{t/T_1}\right) \quad (1)$$

Cell culture - JLat 9.2 T cells

JLat 9.2 T cells (RRID:CVCL_8285, obtained through the NIH HIV reagent program, cat# 9848-69) were cultured in RPMI supplemented with 10 % fetal bovine serum, 2 mM L-glutamine, 10 U/mL penicillin and streptomycin and 20 mM sodium pyruvate and grown at 37 ^{o}C with 5 % CO_2 in a humidified incubator.

Cell culture - HEK 293T cells

HEK 293T cells (CVCL 0063, obtained from ATCC cat# CRL-3216) were cultured in DMEM supplemented with 10 % fetal bovine serum, 2 mM L-glutamine, 10 U/mL penicillin and streptomycin and 20 mM sodium pyruvate and grown at 37 ^{o}C with 5 % CO_2 in a humidified incubator. For isotope labelling, 1 x 10^5 cells were seeded into T75 flasks and grown with ^{13}C,^{15}N-Bioexpress media (Cambridge Isotopes) supplemented as described above and grown to confluence.

DNP Sample preparation

JLat 9.2 T cells were prepared for DNP NMR by removing the culture media and washing the cells with deuterated PBS. For preparation with AsymPol-OlyA, 40 million washed cells were resuspended in freshly prepared AsymPol-OlyA at a molar ratio of 1 nmol/million cells and incubated for 15 min on ice. The excess AsymPol-OlyA was removed, d_6-DMSO added to a final concentration of 10 % v/v and the cells were pelleted into a sapphire 3.2mm rotor at 1500 rpm for 1 min and then flash frozen in liquid nitrogen. For the preparation of cells with AsymPol-POK, 40 million washed cells were mixed with AsymPol-POK to a final concentration of 5 mM and d_6-DMSO added to a final concentration of 10 % v/v. The cells were then pelleted into a sapphire rotor as described above.

EPR spectroscopy

For EPR experiments, 30 μL of protein sample were transferred into glass capillaries with 0.9 mm outer diameter (Micropipettes, Blaubrand, VWR). Spectra were recorded on either a Bruker ElexSysE500 X-band (9.9 GHz) CW EPR spectrometer (AsymPol-OlyA sample) or a Bruker EMX X-band (9.9 GHz) CW EPR spectrometer (AsymPol-M4-SDSL sample) equipped with a Super-High-Q resonator (Bruker) at 100 kHz B-field modulation frequency and 0.1 mT B-field modulation amplitude. A receiver gain of 42 dB, attenuation of 23 dB (yielding 1.008 mW incident microwave power), a receiver conversion time of 81.92 ms, and a lock-in time constant of 20.48 ms were applied over 8 scans with 2048 points. Spin-labeling efficiency was determined via digital double integration with baseline correction in MATLAB utilizing the EasySpin software package[46] and referenced to a 4-Oxo-Tempo standard solution of known concentration.

Confocal microscopy

2.3 × 10^6 JLat 9.2 T cells were washed with phosphate buffered saline (PBS) and incubated for 10 min in 75 μL of 15 μM AF647-OlyA (equating to 0.5 nmol OlyA/10^6 cells) supplemented with a 1:100 dilution of rabbit anti-GM1 antibody and a 1:1000 dilution of Anti-Rabbit IgG CF568 antibody (Sigma-Aldrich). The cells were washed and supplemented with 10% DMSO before imaging 15 min after incubation. Confocal microscopy was performed with a Nikon NSTORM (Nikon UK, Ltd) system equipped with a Re-scan Confocal Microscope RCM1 (Confocal.nl, Amsterdam, the Netherlands). We used an sCMOS camera (Orca Flash 4.0 V2) and a Nikon SR Apochromat TIRF objective 100x / 1.49 with oil immersion. The different laser excitations were at 561 nm, and 647 nm. The setup was fully controlled, and image acquisition was performed using the NIS-Elements software (Nikon). The implemented re-scan unit provides an enhancement in resolution from 240 nm to 170 nm. Images were processed in ImageJ where the brightness was adjusted such that the brightest pixels had a greyscale value of 50 and the contrast adjusted to make fluorescent structures distinguishable from non-fluorescent structures.

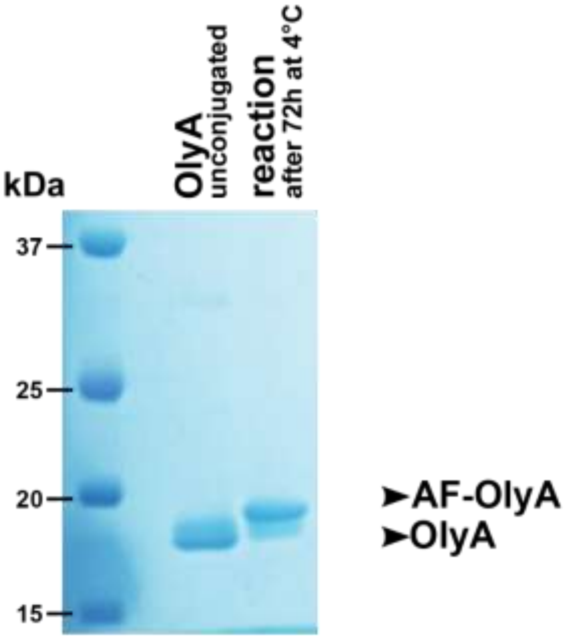

Supporting Information 2: Coupling efficiency of OlyA to AF647. 15% Tris-Tricine SDS-PAGE of purified OlyA and AF647 conjugated OlyA.

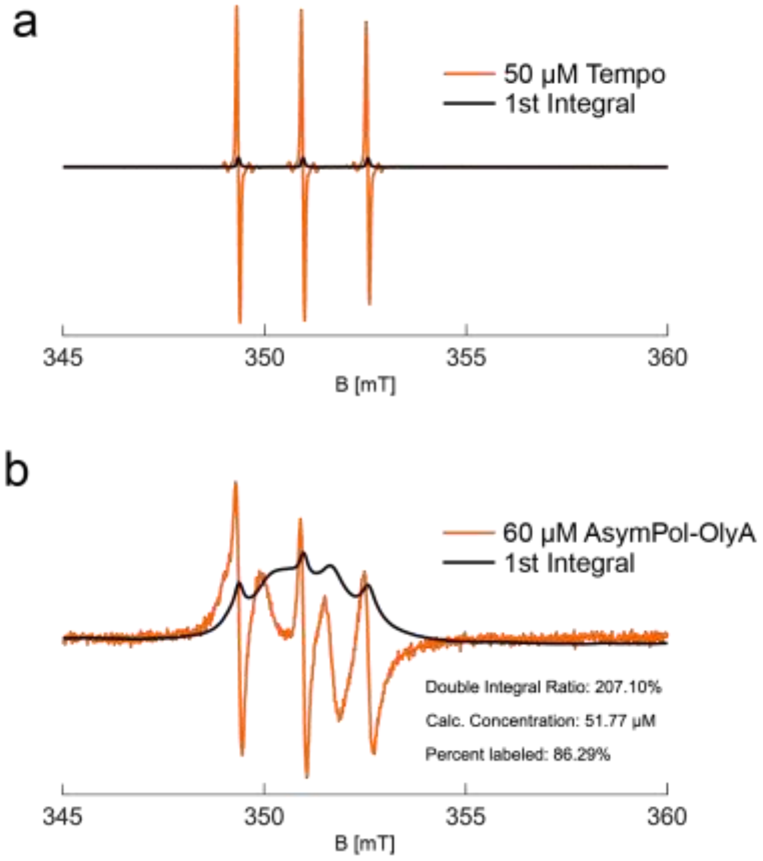

Supporting Information 3: EPR spin counting. EPR spectrum of AsymPol-M4-SDSL (black line) in buffer A and AsymPol-OlyA (orange line) of which 86.3 % was determined to contain radical. 8 scans with 2048 points were recorded on a CW X-band spectrometer.

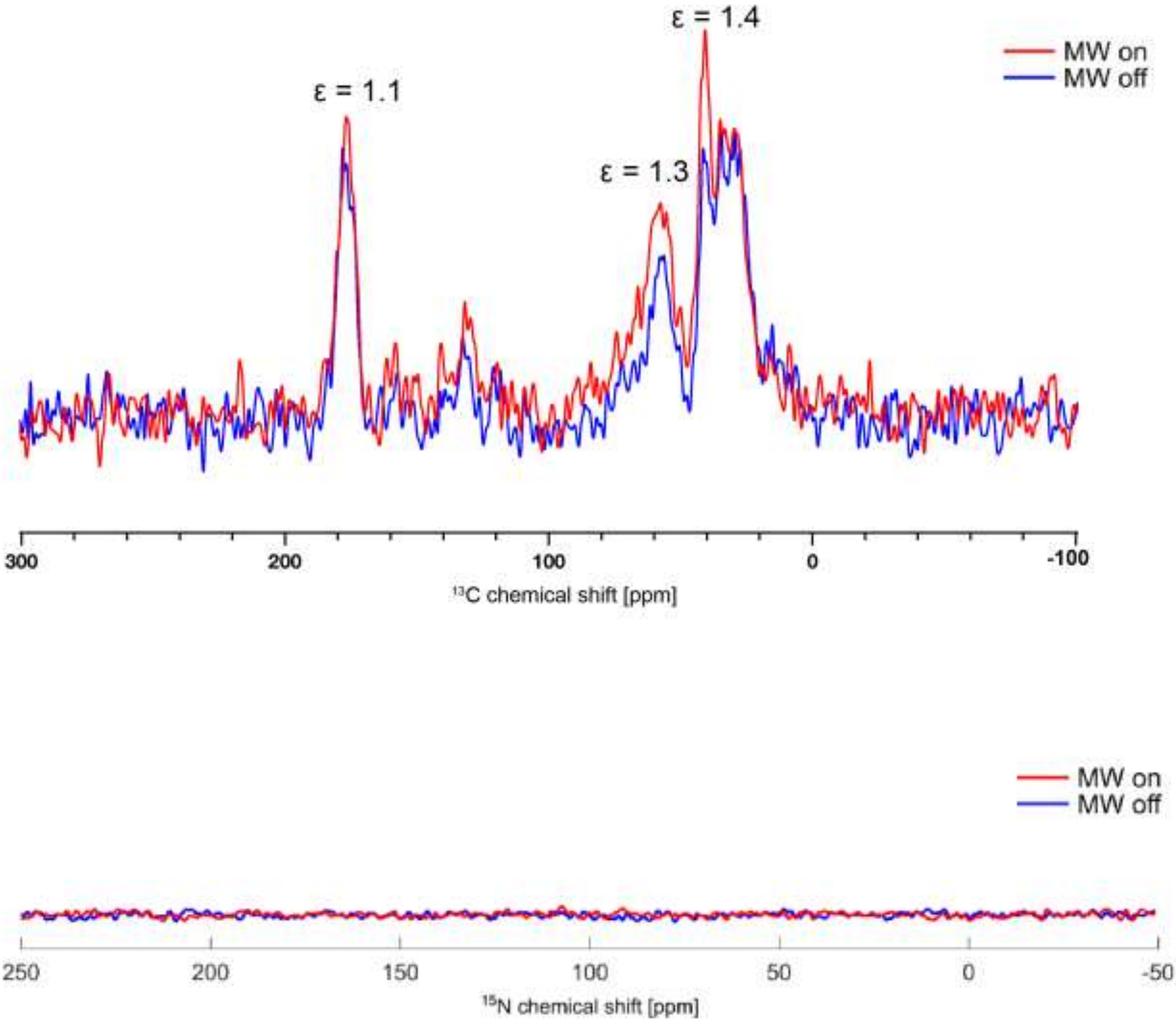

Supporting Information 4: Spectra of ^{15}N-labeled OlyA bound to JLat 9.2 T cells with unlabeled AsymPol-OlyA. JLat 9.2 T cells were labeled with a 1:1 mixture of 0.5 nmol/million cells AsymPol-OlyA: 0.5 nmol/million cells ^{15}N-OlyA (no radical). The free cysteine of ^{15}N-OlyA was quenched with maleimide, reacted under the same conditions as the AsymPol-maleimide radical and purified by size exclusion chromatography prior to labelling. The ^{13}C spectrum is shown on top and the ^{15}N spectrum on the bottom. Data was acquired by cross-polarization with 1540 scans at 9 kHz MAS, 9.4 T external field at 87K microwaves off, 89 K microwaves on with a klystron microwave source with 5.1 W output.
